# Challenges in Treating Older Patients with Acute Myeloid Leukemia

**DOI:** 10.1155/2010/943823

**Published:** 2010-06-10

**Authors:** Lagadinou D. Eleni, Zoumbos C. Nicholas, Spyridonidis Alexandros

**Affiliations:** Division of Hematology/BMT Unit, Patras University Hospital, Patras, Greece

## Abstract

Whereas in younger patients diagnosed with acute myeloid leukemia (AML) treatment is straightforward and the goal is cure, the optimal treatment decision for older adults remains highly controversial. Physicians need to determine whether palliation, “something” beyond palliation, intensive therapy, or an investigational therapy is the most appropriate treatment option. This requires understanding of the biology and risk profile of the AML, clinical judgment in evaluating the functional status of the patient, communication skills in understanding the patient's wishes and social background, and medical expertise in available therapies. The physician has to accurately inform the patient about (a) the unique biological considerations of his leukemia and his prognosis; (b) the risks and benefits of all available treatment options; (c) novel therapeutic approaches and how the patient can get access to these treatments. Last but not least, he has to recommend a treatment. This paper tries to discuss each of these issues.

## 1. Introduction

Elderly acute myeloid leukaemia (AML), generally defined as AML in a patient who is more than 60 years of age,is a clinical entity distinct from the AML in younger adults or children. Unlike in younger adults with AML in which the treatment is straightforward and the goal is cure with intensive chemotherapy, treatment decisions in elderly patients with AML are difficult and remain controversial. Aggressive treatment necessitates hospitalization and separation from family and home, has toxic and potentially fatal side effects, and is often ineffective. There are several factors which influence the treatment decision process. The wishes of patients and their families, performance status, comorbidities, and other less well quantifiable age-related health and social factors are important determinants in the therapeutic decision. Undoubtedly, the advice and influence of physicians has a major impact on treatment decision making [1]. The physician needs to determine whether palliation, “something” beyond palliation, intensive therapy, or an investigational therapy is the most appropriate treatment option. This requires thorough understanding of the biology and risk profile of the AML, clinical judgment in evaluating the functional status of the patient, communication skills in understanding the patient's wishes and social background, and medical expertise and competence in available treatment options and novel approaches. The physician has to give accurate information to the patient about (1) the unique biological considerations of his leukemia and his prognosis; (2) the risks and benefits of all available treatment options; (3) novel therapeutic approaches and how the patient can get access to these treatments. Last but not least he has to recommend a treatment. This review tries to discuss each of these issues.

## 2. Features of AML in the Elderly

AML in the elderly has a grim prognosis. It is of paramount importance to inform the patients and relatives that their disease and their prognosis differ from AML in younger patients. The data of the American SEER-programme (Surveillance, Epidemiology, and End result) report that in comparison to younger patients who have a 30%–35% chance of cure, only 5% of the elderly patients with AML can be cured (http://www.seer.cancer.gov/). Retrospective analyses from haematological centres all over the world and analysis of insurance claims report a median survival that ranges from a few weeks to 4 months irrespective of the treatment given [2–4]. 

Why do older patients fare significantly worse than their younger counterparts? Old age is recognized as a risk factor for both the two major causes of therapeutic failure in AML: treatment related mortality (TRM) and resistance to therapy [5, 6]. Older individuals tolerate less well aggressive therapies due to poor performance status, presence of comorbid disease, decreased ability of clearance of chemotherapy and poor tolerance of systematic bacterial and fungal infections [7]. On the other hand, the disease in older patients shows an increased proportion of unfavorable karyotype (especially abnormalities of chromosomes 5 and 7 or complex chromosomal aberrations) [6, 7], the emergence of AML from an antecedent haematological disorder (AHD) [7, 8], the presence of dysplastic changes [6, 9], the frequent expression of the multidrug resistance (MDR) phenotype [8] and the involvement of more primitive progenitors in the leukemic process [9], all of the above associated with increased resistance to treatment. Recently, a study evaluating gene expression profiling in leukemic samples of 170 elderly AML patients identified subgroups of patients with distinct gene expression signatures [10]. These subgroups also differed in terms of resistant disease, complete remission and leukemia free survival rates, suggesting that gene expression profiling may further shed light on biologic features contributing to the resistance and the adverse prognosis of elderly AML [10].

## 3. Current Available Therapeutic Strategies: Risks and Benefits

Whereas in younger patients the goal of treatment is cure, the optimal treatment decision for older adults with AML remains highly controversial and is a major challenge for clinicians treating these patients. The clinician has to choose from at least four different approaches: supportive care only, less intensive chemotherapy, standard intensive chemotherapy or offering the patient an investigational therapy into a controlled clinical trial [5, 11]. On the one hand, palliation obviously offers the patient no chance for cure. On the other hand, reluctance towards more aggressive approaches relies on the increased risk of TRM due to severe toxicity and the increased costs due to prolonged hospitalization without a clear benefit for the patient regarding overall survival [2, 7]. Further, early results from novel, investigational agents are often promising, however, in many cases they are not confirmed by subsequent studies [5]. Finally, especially in older patients, the benefit has to be determined not only with traditional measurements of outcomes but also in terms of quality of life, control of symptoms, need of transfusions and antibiotics, distance from haematology institution, hospitalisation, and the feeling of independence. The patients should be encouraged to define their treatment goals, in this way actively participating in the decision making process.

### 3.1. Elderly AML: Fit for Intensive Chemotherapy or Palliation?

Intensive chemotherapy in older AML patients is given with the intent to achieve longer life expectancy and possibly a cure. The intensive chemotherapy regime that is most commonly used in elderly AML is the “3 + 7 regime” which is also applied in younger AML patients and consists of 3 days of daunorubicin (45 mg/m^2^ per day) and 7 days cytarabine (100–200 mg/m^2^ per day) [7]. However, older patients exhibit a poor chemotherapeutic tolerance with early death rates ranging from 15% to 25% [5]. Further, the CR rates of these patients are poor, ranging between 30%–55%, these remissions are of short duration and a cure is rarely observed [6]. However, those elderly AML patients who succeed to achieve a CR require less hospitalization and have a relatively good quality of life. In contrast to intensive chemotherapy, palliative care does not aim to cure but includes only supportive measures for the consequences of bone marrow failure caused by AML and/or low dose cytoreductive therapy. The median survival of the patient treated with palliative measures is less than 4 months [2, 4]. Low dose cytarabine is able to induce CR in a fraction of elderly AML patients [12, 13], but it can also induce long lasting myelosuppression and its beneficial effect in terms of overall survival as compared to best supportive care is observed only in the absence of adverse cytogenetics [12, 13]. 

A retrospective analysis of 2657 medicare beneficiaries >65 years old with AML reflects the general attitude of clinicians on the dilemma between palliation or intensive chemotherapy: only 30% of these elderly patients received any intravenous chemotherapy in the 2 years after diagnosis (44% in ages 65–74 years, 24% in ages 75–84 years and 6% in ages >85 years) [2]. Also, Whalin et al., reported on an unselected patient population with AML in northern Sweden that advanced age was a negative predictor for the likelihood of referral to a centre where intensive chemotherapy could be performed [14]. These data suggest that a considerable number of elderly patients are actually offered only palliative care in routine practice. However, emerging evidence suggest that chronological age alone is not adequate to guide the available treatment options [5]. Instead, a “personalized” treatment approach seems to emerge in elderly AML, where therapeutic decisions are individualized based on stratification systems which include (1) the distinctive biologic features of each patient's leukemia as well as (2) efficient assessment of physical status and comorbidity. 

First, the emergence over the last decade of unique cytogenetic and molecular features with a major prognostic impact has defined distinct risk groups among the elderly AML population with almost opposing responses to induction chemotherapy [15]: For instance, elderly patients with favourable cytogenetics (e.g., *t*(8; 21), inv(16)/*t*(16; 16), defined as CBF AML) are probable candidates for intensive chemotherapy due to the high response rates (CR = 88%) reported [16]. In contrast, the likelihood of potential cure with the same treatment is essentially impossible in AML patients with complex or monosomal karyotypes (e.g., those with −7) [5, 17] so as an investigational therapy or palliative therapy is suggested to be offered in these patients instead of standard induction chemotherapy [18, 19]. Importantly, in elderly AML patients with standard risk cytogenetics treatment with intensive chemotherapy results in a median overall survival of up to 12 months, whereas in the adverse cytogenetic category median survival is only 2-3 months, not different from the median survival observed when only best supportive care is offered [15, 20, 21]. Also, additional molecular aberrations within the “intermediate risk” cytogenetic group of elderly AML, as the NPM1 mutation, are shown to provide prognostic information [22, 23]. Importantly, in the absence of leukocytosis (WBC < 50 × 10^9^/L), delaying intensive treatment administration in older AML patients does not seem to have a harmful impact on their outcome regarding CR rates and survival, suggesting that in the elderly population one could probably “wait” for cytogenetic and molecular results to be available to definitively decide the treatment strategy [24].

Second, individuals of the same age are an extremely heterogeneous population, ranging from severely ill to completely ambulatory [7]. Emerging evidence suggest that in elderly AML patients who are extremely aged or have a severely impaired performance status palliative therapy should be preferred. In a retrospective analysis of 968 adults with AML, the combination of a poor performance status (>2-3 ECOG) and advanced age (>75 years) identified a group of patients with a very high likelihood of dying within 30 days of the initiation of induction [25]. Similarly, in another analysis of 998 elderly (>65 years) patients with AML, a poor performance status (>3-4 ECOG) was identified as a risk factor for 8-week mortality and shorter survival [26]. Importantly, in the elderly AML patients with a high level of comorbid disease as defined by a Hematopoetic Cell Transplantation Comorbidity Index (HCTCI) ≥3, median OS after administration of intensive chemotherapy is shown to be similar to the OS observed when only low dose cytarabine is offered [27]. Accordingly, the NCCN guidelines recommend low-intensity therapy or palliation only for older AML patients with an ECOG score >2 [18]. In the same vein, the Italian Society of Hematology guidelines recommends best supportive therapy in patients older than 80 years or with severe comorbidities or with poor and not potentially reversible performance status [19]. In elderly patients with preserved organ function and performance status, palliation is probably not a reasonable option. The European Organization for the Research and Treatment of Cancer (EORTC) formally compared in ambulatory patients older than 65 years intensive induction chemotherapy (daunorubicin 30 mg/m^2^ for 3 days, cytarabine 100 mg/m^2^ for 7 days and vincristine 2 mg) versus a “wait and see” strategy until disease progression followed by palliative therapy with hydroxyurea and subcutaneous aracytin [28]. The median survival was significantly longer in the intensive chemotherapy arm (21 weeks versus 11 weeks) and chemotherapy-treated older patients had a higher chance of survival at 2.5 years (17% versus 0% in the palliative arm). Unexpectedly, there was no difference in days spent in hospital between the two arms. However, this was a small randomized study (31 patients in the chemotherapy arm and 29 in the observation/palliation arm), it was conducted about 20 years ago and in the meanwhile only single-institution or single hospital registry studies addressed the same issues. Recently, a retrospective study by the Swedish national acute leukemia registry compared early death rates, CR rates and overall survival in AML patients aged 70–79 years old from 6 distinct Swedish healthcare regions, which differ according to the therapeutic strategy applied in this elderly patient cohort (distinct proportion of patients offered intensive chemotherapy versus supportive care in each healthcare region) [29]. This study showed improved overall survival in the regions where more AML patients were given intensive treatment and suggested that standard intensive treatment decreases rather than increases early death rates and improves long term survival compared with palliation in elderly patients up to 80 years [29]. These results as well as the results from the EORTC study obviously do not aim to propose standard intensive chemotherapy in elderly AML as a satisfactory therapy. However, they show that standard induction chemotherapy is clearly superior to palliative care for the majority of elderly AML patients, thus putting into question the consistent proportion of elderly AML given only supportive therapy in current clinical practice.

### 3.2. Attempts to Identify the Optimal Induction Chemotherapy Regimen

Considering the disappointing results of standard chemotherapy in elderly AML, a number of groups have evaluated or are currently evaluating various strategies in investigational trials in order to improve outcome. Attempts have been made in order to develop more effective chemotherapeutic regimens with improved tolerability and to reduce drug resistance.

#### 3.2.1. Attempts to Optimize the Standard Induction Regimen

In the beginning, many study groups tried to identify to what extent intensive chemotherapy could and should be performed in older adults with AML, and even if older patients should be treated similarly to younger AML patients. Varying dose intensities of ARA-C and daunorubicin were tested so as to optimize the risk/benefit ratio. Two large multicenter studies performed in England (MRC) and in Germany (AMLCG) reported a significant improvement of survival from full (50–60 mg/m^2^/day) dose versus attenuated dose (30 mg/m^2^/day) daunorubicin without the expense of an increased rate of mortality [30, 31]. More recently, a further escalation of the dose of daunorubicin in elderly AML patients to twice the conventional dose (90 mg/m^2^) showed that in the age group of 60–65 years old the escalated dose of daunorubicin results in improved complete remission, event—free and overall survival rates as compared to standard dose daunorubicin (45 mg/m^2^) without additional toxic effects [32]. Whereas these data demonstrate the importance of intensity and indicate that age should not be a factor for reduction of the anthracyclin dose, this is not the case for cytarabine. Separate randomized trials have found that high dose cytarabine (2-3 g/m^2^) failed to improve survival or CR rate in older patients and often produced toxicity, particularly neurotoxicity [33–35]. Therefore, unlike in younger patients, high dose Ara-C should be omitted in the elderly. Initial reports favoured idarubicin as the best anthracycline to be given in conjunction with cytarabine in the elderly [36]. However, further randomised studies found no difference in long-term outcome between idarubicin, daunorubicin or the anthrachinon derivative mitoxantrone [37]. Other drugs that have been evaluated in elderly AML include 6-thioguanine, etoposide, or fludarabine, mostly in conjunction with an anthracycline derivate and/or cytarabine [37]. Unfortunately, in these studies none of the above regimens seem to offer any substantial survival advantage over the standard regimen [37].

#### 3.2.2. Attempts to Improve Tolerability of the Induction Chemotherapy

The hematopoietic growth factors granulocyte macrophage colony stimulating factor (GM-CSF) and granulocyte colony simulating factor (G-CSF) have been used either to shorten the duration of neutropenia after chemotherapy, or to “prime” leukemic cells so that chemotherapeutic agents might be more effective. The concept of G-SCF priming in AML was based on preclinical studies suggesting that G-SCF and GM-SCF administered prior to chemotherapy induce proliferation of AML cells which sensitizes them to cell cycle–specific agents, as cytarabine [38]. However, a number of clinical trials examining the impact of growth factors administration in elderly AML patients either prior or after chemotherapy did not show any benefit regarding overall survival [39–42]. Nonetheless, the published data support that growth factors' use is safe [39–41], suggesting that their routine use probably rests on economic factors, which is the balance between G-SCF treatment cost and potential G-CSF related decline in hospitalization days. 

Recently, the “priming” concept in AML is reinforced by preclinical studies showing that the SDF-1 inhibitor Plerixafor (AMD3100), clinically developed as a mobilization agent for hematopoietic stem cell transplantation (HCT), sensitizes AML blasts to the effects of chemotherapy due to disruption of their interaction with the bone marrow microenviroment [43]. Results from a phase I/II study evaluating Plerixafor (80, 160 and 240 mcg/kg) prior to salvage chemotherapy in relapsed and refractory AML patients were encouraging, with a CR achieved in 6 out of 8 evaluable patients with no evidence of toxicity [44]. Ongoing clinical trials are evaluating the safety and efficacy of plerixafor as a priming agent in AML.

#### 3.2.3. Attempts to Reverse Drug Resistance

One of the adverse biologic features of elderly AML as compared to younger patients is the more frequent expression of P-glycoprotein, an energy-dependent pump effluxing cytotoxic drugs out of the cell [8]. One strategy to reverse drug resistance that has undergone significant testing in older adults is the adjunctive use in standard induction chemotherapy of agents thought to inhibit P-gP, leading in this way to increased cytotoxic drug retention [37]. Two studies evaluating induction chemotherapy with or without cyclosporine in poor risk AML patients suggested a potential benefit of P-gP modulation in these patients [45, 46]. However, clinical trials evaluating the efficacy of a specific MDR-1 inhibitor PSC-833 (Valspodar) both in younger and elderly AML patients were negative [47–49]. In addition, the more specific MDR-1 inhibitor Zosuquidar was tested in elderly (>60 years) AML patients with standard 3 + 7 induction without any clear benefit [50]. The multiple negative large randomized studies of MRD1 inhibitors in combination with induction chemotherapy in AML so far are discouraging regarding the efficacy of these agents in AML treatment.

## 4. Alternative Treatment Approaches in Elderly AML

As older adults with AML are either judged as “unfit” or do poorly with standard induction chemotherapy, this patient population has created an opportunity to study novel, investigational therapies. However, the decision to offer an older AML patient an investigational therapy should not be based on the promise of the new therapy but on the assessment that, according to the risk stratification and the performance status of the patient, the outcome of standard therapy will be disappointing [5]. A number of novel approaches have entered clinical trials within the last years. There are several new treatment strategies under development, some more and others less toxic:

### 4.1. New Chemotherapies

Clofarabine is a new-generation purine nucleoside analogue which is designed to combine the most favorable pharmacokinetic properties of fludarabine with a less toxic profile [11]. Clofarabine seems to have a significant activity in untreated older adults (>70 years), including those patients with adverse cytogenetics [51, 52]. In particular, clofarabine (30 mg/m^2^ for 5 consecutive days repeated every 28 days) was tested in elderly AML patients (>65 years) considered “unfit” for standard intensive chemotherapy in a phase II nonrandomized study (BIOV-121) [51]. 25% of these patients had unfavourable cytogenetics whereas 55% of patients were >70 years. The overall response rate (ORR) in the unfavourable cytogenetic group was 36% with CR = 27%, while in patients >70 years the ORR and CR rates were 56% and 44%, respectively. Another phase II study evaluating single agent clofarabine (30 mg/m^2^on days 1–5) in 109 previously untreated elderly (>60 years) patients with ≥1 adverse features (high risk cytogenetics, AHD, PS > 2, age >70 years) reported an ORR of 58% in the unfavourable cytogenetic group and 44% in the group with AHD [52]. Further, clofarabine has been tested in combination with low dose cytarabine in previously untreated patients with AML aged 60 years and older. The combination showed a higher CR rate than clofarabine alone (63% versus 31%) with comparable toxicity [53]. 

Cloretazine is a novel DNA alkylating agent selectively targeting the O-6 position in guanine, which is shown to have a significant activity in elderly de novo AML [54] Cloretazine was evaluated as single agent therapy in elderly patients (>60 years) with poor risk AML or high risk MDS [54]. The CR rate in de novo AML was 49% and unexpectedly comparable in the intermediate (50%) and adverse cytogenetics cohort (53%). However, an early death rate of 20% was reported. In a multicenter phase II study a single intravenous infusion of cloretazine (600 mg/m^2^) was tested in 104 previously untreated elderly (>60 years) AML patients [55]. Importantly, no patient had a favorable karyotype whereas a significant cohort of patient had comorbid disease: PS = 2 (30%), pre-existing cardiac disease (45%), and pre-existing hepatic disease (24%). Despite this, ORR was 50% in de novo versus 11% in secondary AML. Response by cytogenetic risk category was 39% in 56 patients with intermediate cytogenetic risk and 24% in 46 patients with unfavorable cytogenetic risk. An early death rate of 18% was reported. Further studies are evaluating the efficacy and toxicity of cloretazine in elderly poor risk AML patients.

Aberrant hypermethylation and silencing of genes involved in cell proliferation and differentiation are commonly found in AML [56]. Therefore, hypomethylating agents may exert antineoplastic activity as inducers of differentiation or response modifiers. 5-azacitidine was approved in 2004 for the treatment of all subtypes of MDS (75 mg/m^2^ given subcutaneously for 7 consecutive days at a 21 days interval) [57, 58]. Recently, the efficacy of azacitidine was compared to conventional care regimens (CCR: best supportive care, low dose cytarabin or standard induction chemotherapy) in a phase III study in 358 patients with higher risk MDS (median age = 69 years) [59]. Interestingly, almost one third of the patients (113 of 358) met the WHO criteria for AML (median bone marrow blasts 23%). Analysis of this patient' subset showed a median overall survival of 24.5 months in the 5-azacitidine arm versus 16 months with conventional care regimens. In addition, 50% of patients treated with 5-azacitidine survived for two years as compared to only 16% of patients treated with CCR. Most common side effects were thrombocytopenia, neutropenia and anemia. These results suggest that the probable survival benefit of 5- azacitidine in MDS may extend to AML patients. More recently, the efficacy of azacytidine was shown in 81 MDS/AML patients with chromosome 5 or 7 abnormalities [60]. 41% patients treated with azacitidine achieved CR, with a median CR duration of 45 weeks. Interestingly, in this single institution study treatment with hypomethylating agents seemed to be superior to chemotherapy [60]. Decitabine is another hypomethylating agent which has been evaluated in AML. A phase II study evaluated the efficacy of low dose decitabine (135 mg/m^2^ repeated every 6 weeks for up to 4 courses) in elderly AML patients (>60 years) not qualifying for, or not consenting to standard induction treatment [61]. So far, results from 278 fully evaluable patients have been reported. Complete and partial remissions occurred in 25% of patients, an antileukemic effect in another 29% whereas early death rate was 13%.

### 4.2. Antibody Targeted Therapies

The surface molecule CD33 is expressed on leukemic blasts of most patients with AML [11]. Gemtuzumab ozogamicin (GO, Mylotarg) is a humanised anti-CD33 antibody linked to a highly potent toxin and is approved since 2001 as the first agent specifically for use in treating older adults with AML (single agent treatment in AML patients ≥60 years at first relapse who are not fit for other cytotoxic therapy) [11]. The approved dose is 9 mg/m^2^ given on days 1 and 15 [11]. Final results of three multicentre open-label single arm phase II studies evaluating GO as monotherapy in the treatment of 277 AML patients at first relapse have been recently reported [62]. 57% of patients were 60 years or older. Patients were scheduled to receive 2 doses of GO (9 mg/m^2^) in a 14–28 days interval. Overall remission in the elderly population was 26%, with CR = 13% and complete response with incomplete platelet recovery (CR_p_) 13%. Importantly, there were no differences in response rates among patients stratified by cytogenetic abnormalities. Elderly patients who achieved CR or CR_p_ had a median overall survival of 11.7 months and 11.4 months, respectively. Grade III or IV neutropenia was a universal side effect observed in 98% of patients; however, sepsis occurred in 18% and pneumonia in 8%. Early death rate in the elderly population was 17%. A neutrophil recovery defined as ≥500/L was observed in a median of 40 days, 43 days, and 51 days from the first dose of GO in the CR, CR_p_, and no response arms respectively. Other side effects included liver function abnormalities, mostly transient and reversible elevations of bilirubin (≥1.5 of the upper limit of normal), whereas 0.9% of patients who received GO and did not undergo prior or subsequent hematopoetic stem cell transplantation developed venoocclusive disease (VOD). Lower dose GO is also being evaluated in relapsed elderly AML in combination with other agents. In this vein, GO (6 mg/m^2^ on day 1 and 4 mg/m^2^ on day 8) and cytarabine (100 mg/m^2^/24 hr on days 1–7) were evaluated in fourteen elderly AML patients with relapsed or secondary AML [63]. Overall response rate was 28%, with two patients achieving CR, one CR_p_ and one partial response (PR). TRM was 28%. In another study 53 elderly patients with poor risk AML (untreated or relapsed/primary refractory) were treated with a combination of GO (6 mg/m^2^ on day 9), cytarabin (100 mg/m^2^/24 hr on days 2–8) and G-CSF (rhG-CSF 5l g/kg on days 1–8); the combination called G-AraMy regimen [64]. The overall response rate was 57% with CR = 43%, CR_p_ = 2% and PR = 21%. 

Further, GO is evaluated as front-line therapy in elderly AML. A study by the GIMEMA/EORTC group evaluated GO (9 mg/m^2^, d1 and d15) in combination with cytotoxic chemotherapy (MICE: mitoxandrone, cytarabine, etoposide) as front line treatment in 57 patients 61–75 years with AML [65]. ORR was 54.4% with CR achievement in 35.1% and complete response with incomplete platelet recovery (CR_p_) of 19.3%. The one year survival in this study was 34%, so this regimen is now being evaluated in a phase III study compared to conventional MICE regimen. Lower dose GO (3 mg/m^2^) has also been studied in combination with other cytotoxic chemotherapies as front line therapy in elderly AML, including fludarabine, cytarabin and idarubicin (My-FlyI regimen) [66] or hydroxyurea and azacytidine [67], with variable results.

### 4.3. Targeted Therapies

Tipifarnib is an orally active selective inhibitor of the enzyme farnesyltrasferase, which modifies proteins for localization to cell membrane [11]. Given that the activity of critical oncoproteins depends on farnesylation, the efficacy of tipifarnib as an antitumor agent has been tested in several tumors, including AML. Although initial results from phase I clinical trials were encouraging [11], subsequent studies failed to show any benefit of tipifarnib treatment compared with palliative care in elderly AML patients [68].

Given the adverse prognosis of FLT3 mutations in younger AML patients, FLT3 inhibition is currently explored as a therapeutic strategy in AML. Several agents with in vitro activity in primary leukemic cells and in vivo activity in rodent models, as lestaurtinib and midostaurin, are evaluated as oral agents in clinical trials [69]. In contrast to younger AML patients, FLT3 ITD do not appear to correlate with an inferior clinical outcome in the elderly patient population, however, a recent phase II trial tested the oral FLT3 inhibitor lestaurtinib (CEP701) as first-line therapy in previously untreated older patients with AML who were not “fit” for intensive chemotherapy [69]. Toxicity was limited and, although there were no CR's, transient reductions in bone marrow and peripheral-blood blasts or longer periods of transfusion independence were observed. FLT3 inhibitors are also being tested in combination with standard chemotherapy.

## 5. Role of HCT in Elderly AML: Allogeneic Hematopoietic Cell Transplantation after Host-Adapted Conditioning

Allogeneic hematopoietic cell transplantation (allo-HCT) can cure patients with myeloid malignancies, however, increased age and frequent lack of sibling donors in older patients discourages the transplantation choice. Therefore, allo-HCT after conventional conditioning has been explored only cautiously in selected older patients [70, 71]. New transplant strategies which incorporate reduced intensity conditioning (RIC) regimens have been established and have been specifically attractive for patients of advanced age [72–86]. Although a detailed discussion is beyond the scope of this review, there are some interesting issues which we have learned over the last years, and others, probably more, which we still “don't know” and are currently under evaluation.

- Allo-HCT in elderly is feasible. A numerous of pilot studies have clearly demonstrated that the goal of reducing transplant related mortality in older patients by using RIC has been accomplished [72, 85, 86].
- Effectiveness. The efficacy of RIC in allo-HCT for elderly patients with AML has not been definitively established because of a lack of controlled trials. Although there are pilot trials reporting very good outcomes in elderly AML patients, allo-HCT is often criticized as being inefficient. However, it must be noticed that there is an overrepresentation of adverse prognostic factors (e.g., complex caryotype, refractory disease etc.) in patients reported in small pilot studies exploring RIC, which might negatively impact any benefit of allo-HCT in controlling leukemia. A lot of variables, both patient as well as transplant specific, may influence outcomes and therefore there is an urgent need to define the optimal transplant strategy in prospective controlled clinical trials.
- Not all RIC regimens are the same [87]. There are a numerous of RIC regimens which vary not only with respect to dose intensity but also to the type of agents used. Since dose does matter [88, 89], most protocols are moving from the initially established “minimal dose” concept with 2 Gy TBI/fludarabin [75, 78] to more intensive, while nontoxic, regimens (reduced toxicity conditioning, RTC). In this vein, the addition of one or two alkylating agents in the conditioning seems to be more effective in controlling leukemia than truly nonmyeloablative ones [79, 84]. Novel drugs like intravenous busulfan preparations [90], treosulfan [91] or clofarabine are promising agents in the field of RIC. Another approach for maximal antitumor activity with reduced toxicity is reported from the Intermountain Blood and Marrow Transplant Program (The LDS Hospital, Salt Lake City, Utah, USA) [92] by using standard, dose-intense targeted busulfan-cyclophosphamide regimen combined with a maximal clinical support. Large randomized studies comparing different regimens are warranted.
- Toxicity and efficacy of a transplant strategy is determined not only from the conditioning regimen but also from the establishment of the donor immune system. A novel late-onset acute Graft versus Host Disease (GvHD) occurring beyond the first 3 months has been observed in the clinical course of RIC [93]. The incorporation of in vivo anti T lymphocyte sera in the conditioning regimen may reduce the incidence of severe GvHD (which is a major concern in the elderly), however, depending on the sera used the risk of opportunistic infections increases.
- Source of stem cells. Although it was initially believed that RIC should be only combined with peripheral blood-derived grafts with their rich supply of effector cells, a recent EBMT retrospective study showed that BM cells can be also used. Elderly patients have very often similar aged siblings which counteract with the safety of stem-cell donation. Nowadays, with improved HLA matching at allele level and efficient GvHD prophylaxis protocols, results from unrelated allogeneic HCT are as good as those after matched sibling-HCT [84, 85, 94].
- The issue as to whether patients with myeloid malignancies, especially the older ones, should receive remission induction chemotherapy before transplantation is not clear. Studies in younger patients with AML suggest that remission status at time of transplantation has a major impact on outcome and therefore a remission induction therapy should be considered before transplantation [95]. However, this seems not to be the case in patients with secondary AML or advanced MDS [96]. It appears that at least for candidates with a smouldering increase of blasts over time, transplantation with a myeloablative, high antileukaemic, age-adapted regimen may be used as front line therapy.
- Epigenetic modulation in the allo-HCT setting. Hypomethylating agents may be used prior to allo-HCT for bridging the time to transplantation or after transplantation either as maintance therapy or to enhance graft versus leukemia (GvL) effect in combination with DLI. Preliminary results of azacytidine given monthly after allo-HCT are very promising [97, 98].

Taken together, age should not be the sole factor for disqualifying a patient as transplant candidate. Some patients, especially those with high risk features, are unprepared to accept a maximal 15% chance of cure with intensive chemotherapy and desire a treatment which has the highest potential for achieving long term survival and cure. Every patient with AML regarded as fit for induction chemotherapy and consented to intensive chemotherapy is also a candidate for allogeneic HCT, should be informed about this treatment option and be referred as early as possible to a transplant center. Comorbidity indexes can help the physician to advice between a transplant or no transplant decision [99].

## 6. Conclusions

AML reaches the highest frequency in the elderly and with the aging of the general population in developed countries, the management of this disorder acquires increasing importance. The optimal treatment decision for elderly AML patients remains a major challenge. The “3 + 7” regimen may be considered standard therapy in older AML patients with preserved performance status and a favorable or standard risk karyotype, however, results remain far from being satisfactory, whereas many older patients, especially those with an unfavorable karyotype, are not benefiting at all from this treatment. Therefore, the best option for elderly AML patients is to be treated within controlled clinical trials. Geriatric assessment tools can help the primary physician with the decision between giving a palliative or curative treatment. First results in allogeneic transplantation after host adapted conditioning are very promising, and indicate that age should not discourage transplantation as a choice.
